# Is a Genetic Diathesis for Poor Nutrition Becoming More Crucial Due to the Uniformity of COVID Social Stress?

**DOI:** 10.3390/nu15040960

**Published:** 2023-02-15

**Authors:** M. Catherine DeSoto

**Affiliations:** Department of Psychology, University of Northern Iowa, Cedar Falls, IA 50613, USA; cathy.desoto@uni.edu

**Keywords:** heritability, gene–environment correlation, nutrition, depression, COVID

## Abstract

The important role of nutrition in proper neural functioning and mental health has seen wider acceptance, but is still sadly under recognized given the existent body of research. This Special Issue was designed to unite authoritative information on this topic in one volume. This editorial provides an overview of the issue, and suggests that the combination of social isolation, lack of exercise, and remaining indoors that overtook industrialized societies during 2020 are specific factors expected to change the Gene × Environment interactions for anxiety and depression. Importantly, the recent environmental changes may make biological diatheses for nutritional deficiencies even more problematic. The concept of G × E interaction is dissected to clarify a non-intuitive scenario: heritability may increase, even when a sharp increase in prevalence is entirely the result of an environmental change (e.g., COVID anxiety and isolation). Key research is highlighted, specific genetic examples are noted, and theoretical implications regarding natural selection are discussed.

## 1. History of Nutrition and Behavior Research

Decades ago, experimental evidence emerged documenting that proper supplementation with vitamins and fatty acids could causally improve behavior. The features of research designs that can properly draw unequivocal cause and effect conclusions include random assignment of the independent variable (nutrients in this case), coupled with quality placebo-control that ensures that intake of the actual nutrients is the only difference between the two groups, thus isolating the causal factor. On the twenty-year anniversary of the first human experimental design investigating nutrition and disruptive behavior in a controlled environment by Gesch and colleagues [1], it is now clear that even when overall diet is monitored, supplementation with key nutrients improves behavior. Prisoners in a facility (all meals standardized) were randomly assigned to receive key nutritional supplements or similar-looking placebo pills, and their behavior was monitored. No side effects were reported, and the prison guards reported a significant reduction in the disruptive behavior of those taking the supplements compared to those taking the placebo. The results were not influenced by participant expectations, nor by the researchers measuring the outcome expectations: the test was fully double-blinded. 

The now classic research above illustrates that the evidence for the role of nutrition in anxiety, depression, and associated behavior is long term, broad and deep. In this Special Issue, Johnson, Shively, and colleagues used non-human primates to achieve the kind of experimental control of dietary conditions that allows clarification of causal statements regarding the effect of nutrition on behavior [2]. The introduction of an unhealthy diet caused a rapid decline in normal behaviors and an increase in anxiety-like behaviors. This finding should feature on the social media feed of everyone who is affected by anxiety or depression. 

Johnson et al.’s study features strong methodology. After six weeks of monitoring baseline behavior while all primates were given the same diet, half were switched to a diet with adequate nutrients and healthy fats, and the other subjects were given a diet that matches a typical modern Western diet in terms of nutritional density. The specifics can be found within the publication, but the poor diet was “designed to mimic the diet typically consumed (by Americans), resulting in a diet rich in saturated fats, sodium, and refined sugars with fats and proteins mostly from animal sources. In contrast, the (healthy Mediterranean-style diet contained) high levels of monounsaturated fats and a lower omega-6:omega-3 fatty acid ratio than the Western diet,” (p. 3, of Johnson et al.). The primates were then monitored for changes in behaviors (self-grooming and scratching, peaceful resting, pro-social affiliation, physical threat charges, displays of aggression, or antagonistic interactions). The research is a must-read for persons who sincerely want to know if nutrition can affect anxiety and behavior. 

In general, the inclusion of whole fresh fruits and vegetables is universally considered important for optimal nutrition and may also impact neural health. Such foods contain myriad co-factors, as well as essential nutrients that humans require, but do not synthesize. The absolute requirement that humans acquire specific nutrients from food—that is sometimes absent from our current diet—makes sense, because natural selection operated under conditions when humans consumed a markedly different diet. Consider that vitamin C is a co-factor in the synthesis of dopamine *β*-hydroxylase (for using and breaking down the reward molecule, dopamine). Moreover, vitamin C is a required co-factor in the conversion of tryptophan into the mood-enhancing serotonin. As noted in this issue [3], problems with dopamine *β*-hydroxylase activity are linked to mood and anxiety disorders. Tryptophan depletion, via intake of concoctions known to specifically and sharply deplete tryptophan [4], has been shown to trigger a drop in mood using a placebo-controlled experimental methodology. Interestingly, the effect may be limited to persons with a genetic code that impacts serotonin reuptake (s versions of the 5-HTTLPR) or in persons with a personal family history of mood disorders—in other words, those with a likely genetically based diathesis [4,5,6].

This Special Issue also features Billows, Kakoschke, and Zajac [3], who highlight two placebo trials that investigate intake of vitamin C via a naturally occurring high C food and overall nutritional powerhouse: the golden kiwi fruit. Both studies show improvement in mood occurred as a result of adding the whole fruits to the diet. In one study by Carr et al., participants were randomly assigned to ingest either two whole kiwi fruits, an amount that would bring their vitamin C levels to optimum (and contains synergistic compounds such as vitamin E and beta carotene), or an amount that mimicked the experience of eating fresh fruit (eating one half of a kiwi fruit) but did not, in fact, come close to providing optimal amounts. Scores on a standard measure of mood improved specifically for those who optimized their circulating vitamin C levels by eating two whole fruits, with the effect being primarily seen in those who had mood disturbance at baseline (potentially a marker of a diathesis). 

A second randomized experiment by a different research group [7] replicated the results. Using placebo control, they also examined the effect with a group (n = 162) with documented low vitamin C at baseline. Participants were randomly assigned to “receive either two gold kiwifruit per day, a daily 250 mg vitamin C supplement or a placebo tablet (1 tablet per day) across the 28-day intervention period,” [3]. Those who consumed the fresh kiwifruits reported significantly less depression and greater well-being. No effects occurred in the placebo condition.

Although positive research findings regarding using food as medicine are rarely mass advertised to the public or highlighted by modern media, given the skyrocketing levels of anxiety and depression, these results seem important to public health. Moreover, the side effects of optimal nutrition are, of course, only helpful.

## 2. Nutrition in the Environment of Evolutionary Adaptation

The importance of any genetic vulnerabilities to nutritional deficiencies is likely new in humanity. The current Western diet devoid of expected nutrient levels and co-factors is novel, and the environment in which natural selection operated for eons is now irrelevant. For example, as detailed by Ortega and colleagues [8], omega-3 fatty acids are critical for neural health. Primate brains are mostly composed of fat. Our fat-built brains are formed based on a genetic code ordering proteins to be produced here versus there—a genetic code that inherently expects not only plenty of fresh fruits and vegetables, but a near one-to-one ratio of omega-3 to omega-6 fatty acid molecules. Today, the genetic code is more likely to encounter neural tissue deficient not only in various co-factors, but that also must function with a 10-fold or greater shift in favor of omega-6 fat molecules [9]. With this in mind, it cannot come as a surprise that at least some minds, at least sometimes, will not function optimally when the expected molecules are absent or deficient. The crowding out of nutrient-dense food by (evolutionarily atypical yet easily acquired) refined sugar and the ubiquitous nutrient-stripped white-flour-based food items is not something our genotypes would be, or could have been, naturally selected to accommodate. 

COVID isolation in the context of diathesis. The social isolation, lack of exercise, and indoor lifestyle that overtook industrialized societies during 2020 are specific factors that have long been predicted to make people more vulnerable to depression and anxiety [9]. That such an increase in the pathology plainly occurred is beyond dispute [10,11]. Many illnesses, including most mental illnesses, occur due to an interaction between heritable and non-heritable factors. This is certainly true of depression and anxiety disorders, and although the heritability estimate for anxiety and major depression varies depending on the exact data used, it has been found to be approximately 35% [12]. To be clear, this means that if heritability is estimated at 35%, that about two-thirds of the variation in who does, and who does not, become anxious and depressed was ultimately due to variations in environmental experiences. The full extent of the ways genetics and environment become intertwined is sometimes not fully appreciated; nor what can happen to the mix if the environmental variance suddenly shifts.

Gene environment interactions. Interaction, as used here, has a meaning of import. This means, among other things, that one’s genotype will cause the body to handle environmental events differently than another. It also means that the environment itself, via experiencing stress or other exposures, impacts how the genome directs the body. Stress and exposures can and do change gene function via DNA methylation and histone modification; persons with different genotypes may exhibit the same gene expression. To really throw a wrench in the outdated idea that nature and nurture are functionally separate influences, note that some of these alterations in genetic function have clearly been passed down to future generations, as if LaMarck was partly right all along; Bohacek and colleagues overview this idea [13]. Inherited genetics will nudge behaviors in ways that will sometimes systematically change environmental exposures, which in turn add to the original vulnerability to further impact phenotype. Although a full discussion is beyond the scope of this editorial, Table 1 briefly summarizes these effects and groups them into three broad types.

Obviously, genetic variations can affect how deficiencies are handled in the brain. However, a perhaps underappreciated fact is that when depressionogenic environments are not encountered, genetic variations that respond to nutritional deficiencies by impacting mood may have little or no effect on the phenotype. For example, as a thought experiment, we can consider that inflammation likely affects multiple processes, including methylation and histone modification [14], turning genes on or off, and the individual level of inflammation is itself directly affected by both an individual’s genetic code and environment. If Person A has little inflammation due to a generally healthy diet, her genes may function like those of Person B; but if her diet is pro-inflammatory, for example, and her gut biome is affected, whilst her vitamins C and D levels are low, her body may respond to the stress by methylating some genes, which then function differently. Moreover, the genes and behavioral tendencies associated with A’s genotype also impact the environment via behavioral nudges. For example, genes that predispose to inhibition may result in choosing greater social isolation, more time spent indoors, and less Vitamin D. If so, this would be a primary, active choice (Type III) E × G interaction. The lesser Vitamin D may itself further promote inflammation and affect mood via this inflammation, as well as neural effects associated with nutrient status. However, if A’s diet was non-inflammatory to begin, with a fresh food intake and omega 3 to 6 ratio similar to that of humans in previous centuries, the lack of vitamin D caused by being indoors, even if partly caused by her inhibition genes, would not result in a mood disorder. Genetic and environmental influences do not stay separate. 

As noted by Dunn and colleagues [15], the field suffers from a lack of uniformity in investigating the nature of G × E interactions. This is a shame because genetics will, and logically must, work to fine-tune how environmental stressors are handled using any available mechanism. Such effects come in at least three types. Type 1 is G × E interaction, which occurs via a specific gene’s effect on central nervous system functioning (e.g., how a cell reacts to a lack of oxygen, or how receptors handle a neurotransmitter at the synapse), as well as via genetics that impart tendencies to make behavioral choices—choices that then directly affect the environment encountered (e.g., preference for sugar, preference to socially isolate). This, in turn, may affect the physical functioning of the individual, leading to a Type II E × G interaction. Finally, there are environmentally triggered epigenetic effects on gene expression outside the A-T and G-C nucleotide coding (Type III).

Genetic variations related to nutrition can become more important. Some individuals, who lack a genome-based diathesis, will be largely immune to the stress of nutritional deficiency, even when the environments are highly conducive to the spread of disease (e.g., the relatively recent COVID isolation and remaining indoors promoting anxiety and depression). It is important to realize that, if so, then the importance of genetics will likely have increased, even when a sharp increase in prevalence is all due to environmental change. 

For clarity, it can be considered that five years ago, only some people existed in a depression-promoting environment (lacking regular exposure to daylight, low to no physical activity, and little to no social interactions) along with the Standard American Diet. In 2020, some of this environmental variation suddenly and sharply lessened. Suddenly, virtually everyone was living in a depression- and anxiety-provoking environment [9]: The range of environmental variation lessened. Although in biology [16], it has long been taken for granted that changes in environmental variation will change estimates of heritability (e.g., that V_phenotype_ = V_genes_ + V_environment_), this may be less clear among psychologists and therapists who treat mood disorders. One might imagine, in 2015, a set of Monozygotic twins—call them Person A (Ann) and Person B (Beth)—who may have significant differences in their environment. Ann might sprain her ankle (a non-shared environmental event), causing her (only) to stay inside and miss track season; combining this with the other depression factors and genotypic diathesis, Ann develops a mood disorder, while Beth, who does participate in track, does not. Contrast to 2020: now Ann with her ankle sprain and Beth without both stay inside and isolate, and they both get depressed. Extending variations of such scenarios to the population, we can see that as environmental differences lessen, the importance of genetic variations will increase. A 2021 versus 2015 twin study calculating heritability might document this, and research to investigate the possibility is needed. The specifics are an aside, but the point is that G × E is real, genetics and environment do not exist as separate influences, and population-level environmental shifts can increase the importance of genetic vulnerabilities.

**Table 1:** Three Ways Nature and Nurture are Not Separate Influences.

Type and Associated Nomenclatures	Possible Examples	References
**G × E type I** Gene and Environment interaction	Low intake of tryptophan may trigger a drop in mood, with the effect limited to persons with a genetic code that impacts serotonin reuptake (s versions of the 5-HTTLPR gene). Vitamin D blood level and polymorphism of rs117102029 may interact in predicting the level of depression. The relationship between depression and vitamin D in the blood is different based on the genetics of an individual.	Neumeister et al. [4] Zhang et al. [17]
**G × E type II** Gene-environment correlation, rGE, evocative and active interactions, gene environment covariation	TPH2 (SNP G versus T) polymorphisms predicts harm avoidance and CNTNAP2 gene (SNP A versus G allele) may predispose to social interaction anxiety. These genes may be also associated with eliciting experiences and harsh interactions that may themselves be depressionogenic compared to those with the other genotypes.	Rutter et al. [18] Stein et al. [19] Rutter et al. [20] Scarr and McCartney [21]
**G × E type III** Epigenetic modification, histone modification or methylation as a result of experience Transgenerational	FKBP5 gene encodes a protein important in the negative feedback loop of the HPA axis and is polymorphic: Persons with the SNP T rather than C are at greater risk for developing PTSD via the increased activity of the gene. BUT—high stress during childhood results causes methylation that increases the activity of FKBP3, independent of the DNA coding. Two different genotypes may function identically depending on the environment encounters. In a mouse model, classically conditioned fear can cause odor-receptor genes to be methylated in sperm cells, with the conditioned fear then appearing in the subsequent generation even without the conditioning experience.	Klengel et al. [22] Dias and Ressler [23]

It is reasonable to hypothesize that during mass social isolation, nearly anyone who has a tendency to become anxious and depressed in the face of generally poor nutrients would then do so. During 2020, if a person had the diathesis, they would succumb, whereas five years prior, a percentage were protected due to the variations in environment. Novel environmental challenges can cause formerly largely neutral genetic variations in dietary factors to suddenly result in a sharp lack of fitness [24,25].

## 3. Conclusions

Experimental research is unequivocal: primate bodies do not work optimally when a modern Western diet is consumed. Just as a poor diet increases body weight, insulin resistance, and fatty liver disease [26], diet has been shown to effect neuroanatomy and affect phenotypic mental health [27]. In all cases, having a genetic vulnerability to poor nutrition is likely to matter only if nutrition intake is poor and to affect mood primarily when the environment encountered is otherwise depressionogenic. The current Special Issue focuses on current documentation of the links between mood and nutrition. The effect on the gut microbiome is becoming well documented, as noted by Ullah and colleagues [28], and the connection between gut and brain health has become increasingly clear, as discussed by Chojnacki and colleagues [29]. At this point, the causal effect of diet on anxiety, depression, and associated behaviors is unambiguous, and should be undeniable.

As editor, having read the works of the contributing authors, I am struck afresh at the strength of the data that document nutritional effects on depression and anxiety. A belief that the diet—fundamentally the intake of the required neuronal messaging system building blocks—is irrelevant to mood disturbance and anxiety is adding to suffering. If anxious and depressed individuals are to make decisions based on science and data, considering the actual state of the full body of scientific literature (anecdotal, correlational, longitudinal, and placebo-controlled experimental designs, with human, other primates, and mouse models; at the level of the neuron and the gut; observable and documented both in vitro and in the behaving animal), they will consider the full body of science: what natural selection is, under what circumstances it has wrought a brain and body to optimal functioning, and how biology builds neural networks that guide behavior. Those struggling with anxiety and depression, and those seeking to help them, must come to terms with the fact that diet and nutrient intake has drastically changed, and not for the better. Then, perhaps, they will once again understand that food and nutrition can, and must be, considered as part of the puzzle.

This Special Issue is dedicated to you.
